# Supplementary material for: Patient coaching: What do patients want? A mixed methods study in waiting rooms of outpatient clinics
Source: PLoS One. 2022 Jun 9;17(6):e0269677. doi: 10.1371/journal.pone.0269677 (PMC9182226; doi:10.1371/journal.pone.0269677)
Supplement: S1 Appendix — (PDF) [file pone.0269677.s001.pdf]

Appendix I

Questionnaire waiting room study

Code:
Outpatient clinic
Date:

Below are some questions about your situation:

<u>Question 1</u>

are you:

- *Male?*
- *Female?*

<u>Question 2</u>

What is your age? ……..

<u>Question 3</u>

Do you read and speak Dutch?

- *Yes*
- *No*

<u>Question 4</u>

Where do you currently live? ……..

<u>Question 5</u>

Do you have a chronic illness? If so, what kind of chronic illness? (You may also indicate more, underline the most important ones for you):

- *Yes, being ……….*
- *No*

<u>Question 6</u>

Who did you come here with?

- *Alone*

- *With my spouse*
- *With my child*
- *With my friend*
- *With a professional companion*
- *Other, being……….*

Question 7

What is your highest level of education? (completed a study program with diploma or sufficient certificate)

- *No education (primary education: not completed) - primary education (primary school, special education)*
- *Primary or prepared vocational education (such as LTS, LEAO, LHNO, VMBO)*
- *Middle general secondary education (such as MAVO, (M) ULO, MBO short, VMBO-t)*
- *Secondary vocational education and vocational guidance education (such as MBO-long, MTS, MEAO, BOL, BBL, INAS)*
- *Higher general and pre-university education (such as HAVO, VWO, Atheneum, Gymnasium, HBS, MMS)*
- *Higher professional education (HBO, HTS, HEAO, HBO-V, Candidate university education)*
- *Scientific education (university)*
- *Other, being ……………………………………………….*

The following questions are about how you generally communicate with doctors as a patient.

For each question, please indicate how much confidence you have that you will be able to do this?

If you have no confidence at all, tick the leftmost box. The more confidence you have in it, the more to the right you tick the box.

	No confidence at all	Little confidence	Not much / not little	Fair confidence	Lots of confidence
1. Know what questions to ask him / her?					
2. Are you able to have him / her answer all your questions?					

3. Make the most of the visit to him / her?					
4. Are able to make him / her your most important health complaint to be taken seriously?					
5. Are you able to get him / her to do something about your main health complaint?					

Finally, whether you would be interested in a patient coach:

Question 8

Would you need a coach?

- *Yes, why? ….*
- *No, why not?….*

If you answered "yes", and if you are willing to participate in an interview, you may write your contact details at the bottom of the page. You will be contacted within 2 weeks for an interview.

If you answered "no", would you please hand in the form at the return point at the desk or at one of us?

Thank you for completing this questionnaire.

I am willing to participate in an interview of approximately 1 hour, in a place that suits me. Prior to that, it is explained to me what the intention is. At that moment I can still decide not to participate.

I can be contacted using the information below:

Name:

Phone number:

Email:
