# Supplementary material for: Patient coaching: What do patients want? A mixed methods study in waiting rooms of outpatient clinics
Source: PLoS One. 2022 Jun 9;17(6):e0269677. doi: 10.1371/journal.pone.0269677 (PMC9182226; doi:10.1371/journal.pone.0269677)
Supplement: S2 Appendix — (PDF) [file pone.0269677.s002.pdf]

Appendix II

Demographic data code:

Date interview:……………………………………………………………………………

Place interview:…………………………………………………………………………

Name interviewer:………………………………………………………………………

This therefore concerns conversations with your most important medical specialist in which you have indicated that you would be interested in a coach who helps you with the preparation, who will accompany you to the meeting and discuss the conversation with you.

The interview will take place based on a subject list, but I would like to write down some of your details first.

Male / Female

Date of birth:……………………………………………………………………………

Living situation: (with partner, children, alone etc.)……………………………………

Highest level of education? (with certificate) ………………………………………….

Country of birth of your own?…………………………………………………………….

Country of birth of your mother? ……………………………………………………….

Country of birth of your father?…………………………………………………………

Chronic illness (most important)………..………………………………………………

Illness duration: …………………………………………………………………………….

Other chronic diseases? ………………………………………………………………….

Existing for how long? …………………………………………………………………….

Number of medicals specialists involved?……………………………………………..

Number of contacts with most important medical specialist per year?………………

How long with this medical specialist?…………………………………………………

Do you usually go alone or with someone?……………………………………………

With whom? ………………………………………………………………………………

Signed informed consent?

Topiclist patient coaching

Describe patient situation.

a. Does it concern one specialist, or several with whom you would be interested in a coach, does this depend on the specialist? How come you experience differences in the consultations? If there are several people to whom it would apply: discuss one by one.

b. Discuss experiences, what barriers do you experience? How have you solved that so far?

c. Why would you want a coach to accompany you? What do you think a coach can do differently?

What could a coach do for you?

a. Think of the 5 W's. WHAT could such a coach do? What would help you? WHERE would you meet the coach and WHEN, at what times, how often should the coach come along, etc.

b. Where should the emphasis be in terms of guidance: practical, substantive, emotional, or a combination, or something else? What is (most) important? And why?

Who might offer this kind of support?

What kind of person is that? (the point is to keep thinking in terms of personal guidance). Also try to make this as clear and imaginable as possible, in order to arrive at a kind of profile.

Examples of barriers that people experience (as a warming-up):
People sometimes find it difficult to speak with a medical specialist for various reasons. What reasons apply to you? Because….

1. Are feeling tense
2. Are burdensome to talk about the subject
3. Are looking up to the provider
4. Are feeling embarrassed about the subject
5. Feel uncertain about own understanding
6. Are Expecting an annoyed/offended response from provider
7. Perceive there is too little time
8. Believe subject is not important enough
9. Do not know how to discuss the subject
10. Remember the subject only afterwards
11. Do not find the right moment to bring something up
12. Do not want to be bothersome
13. Do not know what to ask
14. Fear the answer to their question

15. Believe provider cannot provide solution/answer anyway
16. Believe subject is not part of this providers task
17. Do not know what is expected from them
18. Other:

These barriers were also used in previous research (Henselmans, 2014) among people with a chronic disease, such as rheumatism, cancer, COPD. This showed that 1 in 6 people with a chronic condition would like a coach to join a meeting with a medical specialist.
