# Supplementary material for: Patient coaching: What do patients want? A mixed methods study in waiting rooms of outpatient clinics
Source: PLoS One. 2022 Jun 9;17(6):e0269677. doi: 10.1371/journal.pone.0269677 (PMC9182226; doi:10.1371/journal.pone.0269677)
Supplement: Attachment — Submitted filename: 20220428 Response to reviewer #2, Alders et al.docx [file pone.0269677.s004.docx]

Zwolle, April 28^th^, 2022

Dear mrs. Boehmer,

Thank you for your remark. We added information which distinguishes a patient coach from a Board Certified Health and Wellness coach in the abstract and in the text. We trust these adjustments are acceptable to you.

The abstract now reads as:

Lines 26 -27: “These patients could benefit from support from a coach who accompanies them to and during medical specialist consultations to improve communication in the consultation room.”

The text in the introduction now reads as:

Lines 86 – 90: “We defined the concept of patient coaching as personal support for patients, aiming at improving communication in consultation with a medical specialist. The patient is supported in the preparation of the consultation, accompanied during the consultation and in the evaluation of the consultation with a medical specialist afterwards.”

Kind regards,

On behalf of the authors,

Irène Alders
